# iTRAQ-based quantitative proteomic analysis of the improved effects of total flavones of *Dracocephalum Moldavica* L. in chronic mountain sickness

**DOI:** 10.1038/s41598-021-97091-z

**Published:** 2021-09-01

**Authors:** Aihaidan Abudouwayiti, Yiliyaer Nijiati, Xiangyang Zhang, Dilinuer Maimaitiyiming, Ainiwaer Aikemu

**Affiliations:** 1 The First Affiliated Hospital of Xinjiang Medical University, No. 1, Liyushan Road, Xinshi District, Urumqi, 830054 Xinjiang China; 2 Central Laboratory of Xinjiang Medical University, Urumqi, 830011 Xinjiang China; 3 Department of Comprehensive Cardiac Medicine, The First Affiliated Hospital of Xinjiang Medical University, Urumqi, 830054 Xinjiang China; 4 Department of Drug Analysis, College of Pharmacy, Xinjiang Medical University, Urumqi, 830017 Xinjiang China; 5 Key Laboratory of Active Components of Xinjiang Natural Medicine and Drug Release Technology, Xinjiang Medical University, Urumqi, 830017 China

**Keywords:** Biotechnology, Biomarkers, Cardiology, Diseases

## Abstract

To use isobaric tags for relative and absolute quantification (iTRAQ) technology to study the pathogenesis of chronic mountain sickness (CMS), identify biomarkers for CMS, and investigate the effect of total flavones of *Dracocephalum moldavica* L. (TFDM) on a rat model of CMS. We simulated high altitude hypobaric hypoxia conditions and generated a rat model of CMS. Following the administration of TFDM, we measured the pulmonary artery pressure and serum levels of hemoglobin (Hb), the hematocrit (Hct), and observed the structure of the pulmonary artery in experimental rats. Furthermore, we applied iTRAQ-labeled quantitative proteomics technology to identify differentially expressed proteins (DEPs) in the serum, performed bioinformatics analysis, and verified the DEPs by immunohistochemistry. Analysis showed that the pulmonary artery pressure, serum levels of Hb, and the Hct, were significantly increased in a rat model of CMS (*P* < 0.05). Pathological analysis of lung tissue and pulmonary artery tissue showed that the alveolar compartment had obvious hyperplasia and the pulmonary artery degree of muscularization was enhanced. Both pulmonary artery pressure and tissue morphology were improved following the administration of TFDM. We identified 532 DEPs by quantitative proteomics; gene ontology (GO)and Kyoto Encyclopedia of Genes and Genomes (KEGG) pathway analysis further revealed that metabolic pathways associated with coagulation and complement play crucial roles in the occurrence of CMS. Immunohistochemistry verified that several DEPs (α-1-acid glycoprotein, collagen, fibulin, haptoglobin, PLTP, and TAGLN2) are important biological markers for CMS. Our analyses demonstrated that TFDM can improve CMS and exert action by influencing the metabolic pathways associated with coagulation and complement. This process relieves pulmonary artery pressure and improves lung function. We also identified that α-1-acid glycoprotein, collagen, fibulin, haptoglobin, PLTP, and TAGLN2 may represent potential biomarkers for CMS.

## Introduction

Chronic mountain sickness (CMS) is a clinical syndrome caused by the influence of a high-altitude hypoxic environment. CMS often occurs in people living in high-altitude areas above 3000 m or long-term immigrants. CMS is a plateau disease that is caused by dysfunction in the human body’s compensatory actions to a chronic hypoxic environment at altitude^1^. The main feature of CMS is an excessive increase in red blood cells, with or without pulmonary hypertension. The pathogenesis of CMS is not yet clear, although it has been established that a high-altitude hypoxic environment is the main reason for its occurrence^2^. Globally, more than 140 million people live in high-altitude areas above 2500 m; 5% to 10% of these people are at risk of CMS^3^. The current treatment for CMS is mainly symptomatic. The current treatment for CMS is mainly symptomatic. The most effective practice in managing the condition is moving patient permanently to a lower-altitude location, but it is impractical for social, family, and economic reasons. Phlebotomy is another frequent practice that is used to reduce red blood cell mass and Hb concentration, but it will increases pulmonary artery pressure and may aggravate pulmonary hypertension^2^. Several clinical therapies are commonly used to treat CMS in China, including acetazolamide, nifedipine and denosine disodium triphosphate. These have a certain effect on CMS by exerting an antioxidant role in the plasma, improving the function of the heart and lungs, accelerating the programmed death of pulmonary vascular smooth muscle cells, reducing oxidative stress, and by relieving myocardial damage. However, the therapeutic effects of these drugs are not satisfactory; in addition, adverse effects are common^4^.

*Dracocephalum moldavica* L. (DML) is a common medicinal material for Chinese nationals. This medicine is also commonly referred to as Angkailu Mole·Biriyanggu, Badiranjibuya, Moyanzi, mountain mint, blue autumn flower, and corn grass. DML is an annual plant from the labiatae family. DML is used clinically to treat coronary heart disease, hypertension, angina pectoris, arteriosclerosis, myocardial ischemia, and other diseases^5^. Previous studies have found that the active components of *Dracocephalum moldavica* L. (TFDM) can significantly improve hypoxia such as decrease pulmonary artery pressure, and improve the cardiac pathological state without any notable side effects^6^. Previous research studies involving TFDM have mostly focused on pharmacological mechanisms, purification methods, and the analysis of therapeutic effects on humans or animal models^7^.

Animal models are an effective way to study the molecular mechanisms underlying CMS. For example, Dilinuer et al.^5^ placed Sprague–Dawley (SD) rats in a plateau environment laboratory at an altitude of 5000 m. After 45 days, compared with the normal plain control group, the plateau model group showed significant increases in C-reactive protein (CRP), endothelin-1 (ET-1), interleukin-6 (IL-1), homocysteine (HCY), vascular endothelial growth factor (VEGF), hemoglobin (Hb), and hematocrit (Hct), and significant reductions in nitric oxide (NO), oxygen saturation (SaO_2_), and the partial pressure of oxygen (PaO_2)_. Furthermore, cardiac ultrasound showed increases in the inner diameter of the right atrium, the inner diameter of the left ventricle during systole and diastole, and the inner diameter of the right ventricle. The anterior wall of the right ventricle and the ventricular septum showed thickening, the right ventricular outflow tract had widened, ejection fraction had decreased, and the ventricular hypertrophy index had increased. Collectively, these data showed that a rat model of high-altitude sickness had been successfully constructed and showed similar effects as seen in humans.

Over recent years, Liquid chromatography—tandem mass spectrometry (LC–MS/MS) technology coupled with Isobaric tags for relative and absolute quantification (iTRAQ) technology has been used to identify potential early biomarkers of Alzheimer's disease, gastric adenocarcinoma, liver cancer, and acute myocardial infarction^8^. In the present study, we constructed a CMS model in SD rats, administered the rats with TFDM, and used iTRAQ technology to compare proteomic changes. iTRAQ technology was used to identify potential biomarkers and the model rats were used to investigate the effect of TFDM intervention on potential pathogenic differentially expressed proteins (DEPs) in the serum of rats with CMS. Our overall goal was to reveal the pathogenic mechanisms that underlie CMS.

## Results

**Figure 1:**
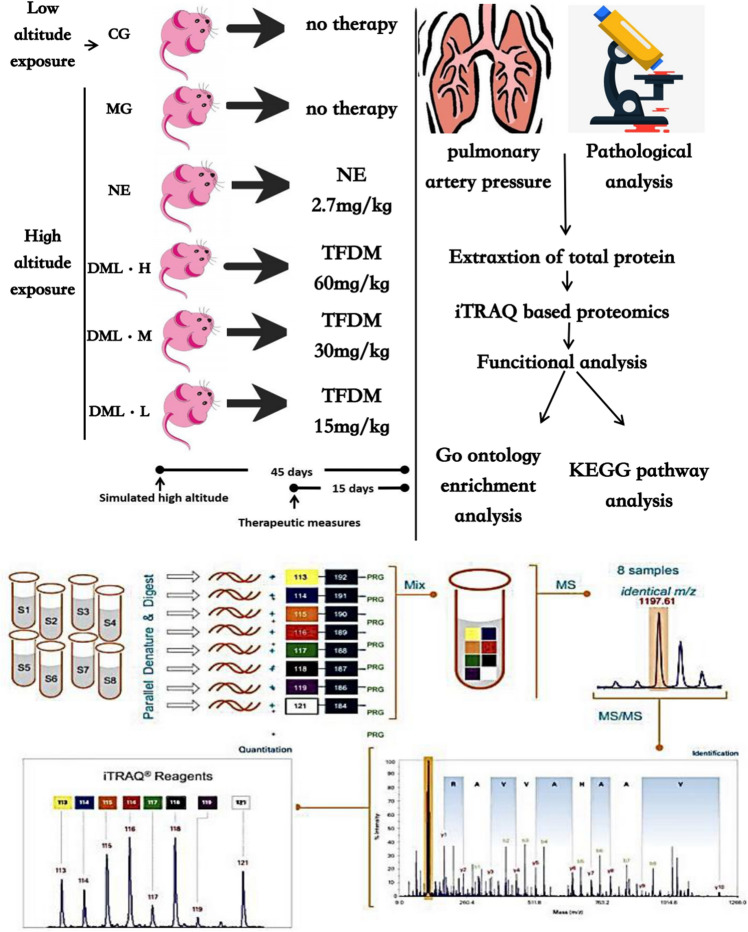
Workflow of iTRAQ analysis in a rat model of CMS. Rats were fed for 45 days under specific experimental conditions. Then, we measured the pulmonary artery pressure, the serum levels of Hb, and the Hct. We also investigated lung tissues and the pulmonary artery. The basic procedure for iTRAQ quantitative proteomics involved several steps: (1) extract protein from the sample, (2) quantify protein concentration, (3) analyze protein samples and evaluate whether the sample quality met the requirements of subsequent experiments, (4) perform reduction alkylation treatment on protein samples that met the quality standards, (5) Take an equal amount of trypsin for each sample and hydrolyze, (6) Label peptides with iTRAQ reagents, (7) mix the labeled peptides in equal amounts, (8) pre-separate the mixed peptides, (9) carry out LC–MS/MS analysis, (10) use the Sequence or Mascot module in Proteome Discoverer to search the database, and (11) perform statistical and bioinformatic analysis using data derived from the database search.

First, we established a rat model of CMS and then performed a series of experiments. After rearing the rats for 45 days in different conditions, we then measured the pulmonary artery pressure, the serum levels of Hb, and the Hct. Furthermore, we also investigated the lung tissue and pulmonary arteries for signs of injury or hypoxia by analyzing changes in tissue structure and function that might lead to remodeling of the lung tissue and vascular system. We found that pulmonary vascular remodeling is an important essential factor that leads to pulmonary hypertension. We used the CMS model to investigate the degree of damage in tissues from the lungs and arterial tissue by analyzing tissue sections of the lungs and pulmonary artery. In addition, we also extracted serum samples from each rat and performed trypsin enzyme digestion. Next, we labeled the peptides with iTRAQ reagent, performed LC–MS/MS analysis, and searched the Rattus database using the Sequence or Mascot module in Proteome Discoverer. Then, we performed statistical and bioinformatic analysis on the search results obtained. The experimental process was shown in Fig. 1.

### A rat model of CMS model was successfully constructed and the administration of TFDM relieved the symptoms of CMS

**Figure 2:**
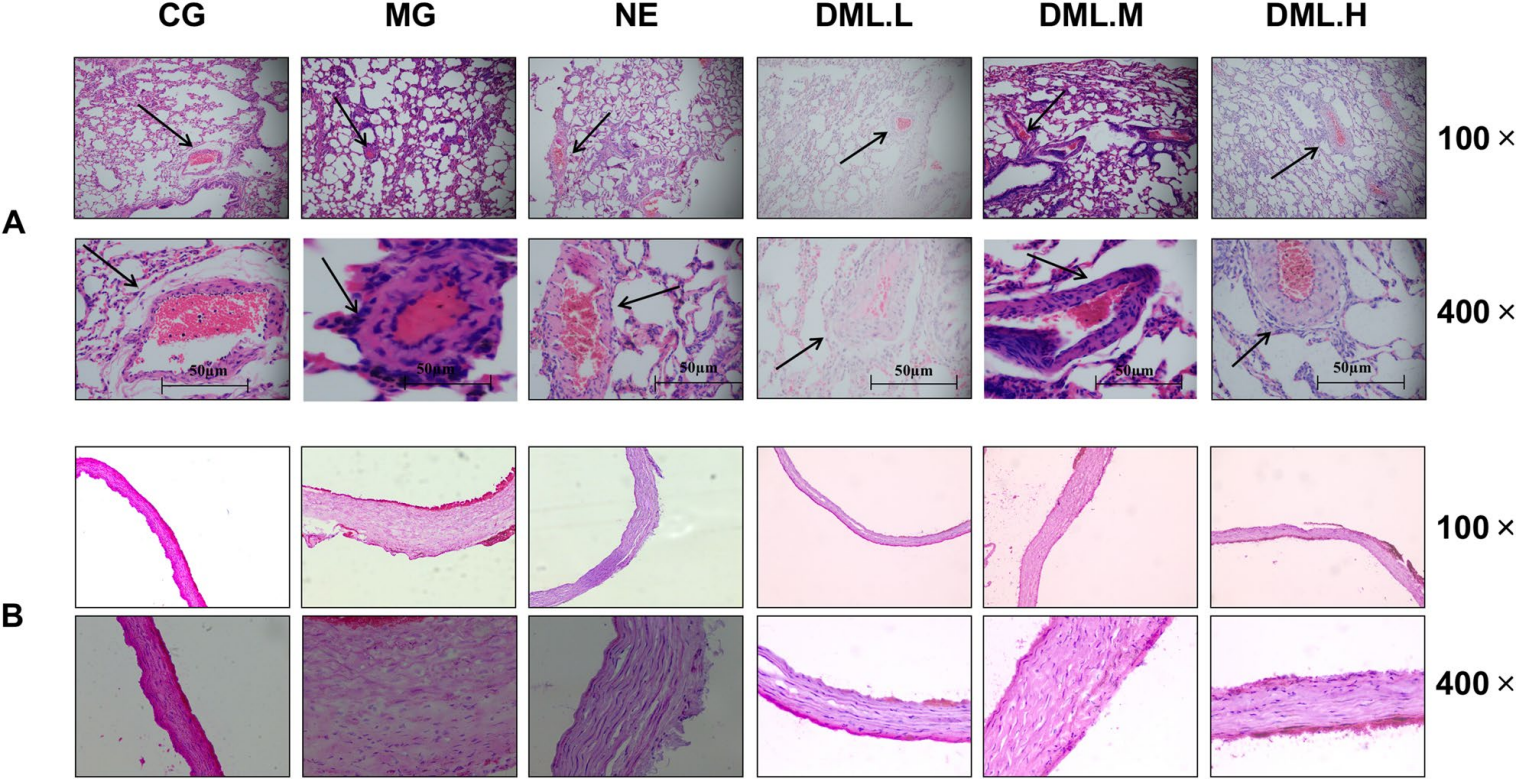
Histological assessment. (**A**) Representative micrographs of H&E staining of the lung tissue (100 × and 400 ×). Arrowheads indicate small pulmonary arteries < 100 µm in external diameter. (**B**) Representative micrographs of H&E staining of the pulmonary artery (100 × and 400 ×).

Following experimental modelling, we found that the plateau model (MG) group had a significantly higher pulmonary artery pressure, serum Hb, and Hct than the control group of rats (*P* < 0.05). In the TFDM experiments, we found that as the dose increased, the pulmonary artery pressure, the serum levels of Hb, and the Hct, all decreased accordingly; as shown in Table 1. Hematoxylin and eosin (H&E) stained images of the lung and pulmonary artery tissue showed that the lung tissue structure in the plain control (CG) group was complete (Fig. 2A), the alveolar wall was thin, the alveolar cavity was clear. Rats in this group also showed some thickening of the alveolar septa, although there were notable pathological changes, and no abnormalities such as alveolar septal hyperplasia and congestion. In the MG group, the alveolar compartment had obvious hyperplasia, the alveolar blood vessels were dilated and congested, and there was exudate and congestion in the individual alveolar cavity. We also observed pulmonary interstitial edema with inflammatory cell infiltration. Following the administration of TFDM, the lung tissue showed thin alveolar walls, slight thickening of some alveolar compartments, a clear alveolar cavity; the lesions was slightly lighter than that of the middle-dose group without other abnormalities. Table 2 shows data relating to the vessel dimensions of pulmonary arteries; both WT and %WT were significantly thicker in the MG group than in the CG group. The WT and WT% of the total flavones of Dracocephalum Moldavica L. low-dose group (DML-L) group were closest to those of the CG group. As shown in Fig. 2B, under the light microscope, the pulmonary artery wall of the CG group was intact, the structure was typical, and the blood vessel walls were clear. The MG group had the most significant morphological changes: the pulmonary artery wall had obvious hypertrophy, and the endothelial cells of the pulmonary artery intima had obviously increased. There was also hyperplasia, the structure of the elastic fibers of the media was disordered, the fibroblasts in the adventitia had thickened, and the degree of muscularization was enhanced. Following the administration of TFDM, the morphology of the pulmonary artery tissue had improved to differing degrees; such improvement was most obvious in the TFDM low-dose group (DML·L). Under low magnification, the pulmonary artery wall showed thickening. Under high magnification, the intimal endothelial cells showed proliferation, the median elastic fibers had better continuity, and the outer membrane fiber cells had slightly thickened.

**Table 1:** The effects of DML treatment on pulmonary artery pressure, the serum levels of Hb, and the Hct, of rats in each group (mean ± D).

Group	n	Pulmonary artery pressure	Hb (g/L)	Hct (%)
CG	20	18.92 ± 2.08	164.44 ± 7.62	33.4 ± 1.9
MG	20	40.13 ± 2.36*	256.78 ± 16.63*	62.3 ± 4.7*
NE	20	29.08 ± 2.69*^Δ^	243.01 ± 17.44^*#^	54.5 ± 3.7*^#^
DML·L	20	36.11 ± 3.21*^Δ^	223.45 ± 11.24*^#^☆	50.4 ± 3.4*^#^☆
DML·M	20	33.15 ± 2.87*^Δ^	215.01 ± 10.45*^#^☆	49.7 ± 3.4*^#^☆
DML·H	20	32.08 ± 1.89*^Δ^	199.24 ± 12.23*^#^☆	45.1 ± 1.1*^#^☆

Data are expressed as means ± standard deviation (n = 20 per group). Plain control group-CG; CMS group-MG; Nifedipine group-NE; Dracocephalum moldavica L. low dosage group- DML·L; Dracocephalum moldavica L. middle dosage group- DML·M; Dracocephalum moldavica L. high dosage group- DML·H; hemoglobin- Hb; Hematocrit- Hct.

*Significantly difference from the CG group.

*P < 0.05, Δ significantly different from the MG group, Δ P < 0.05.

**Table 2:** Vessel dimensions of muscular pulmonary arteries in each group (mean ± SD).

Group	n	External diameter (µm)	WT(µm)	%WT (%)
CG	20	92.7 ± 23.4	10.48 ± 2.8	22.6 ± 3.3
MG	20	89.8 ± 18.0*	16.16 ± 4.5*	36.0 ± 4.3*
NE	20	90.2 ± 18.7*Δ	13.62 ± 4.3*^#^	30.2 ± 2.6*^#^
DML·L	20	95.7 ± 11.7*Δ	12.78 ± 4.3*^#^☆	26.7 ± 1.7*^#^☆
DML·M	20	92.6 ± 10.2*Δ	12.69 ± 3.9*^#^☆	27.4 ± 2.8*^#^☆
DML·H	20	94.5 ± 8.9*Δ	14.08 ± 4.2*^#^☆	29.8 ± 3.0*^#^☆

Data are expressed as means ± standard deviation (n = 20 per group). Plain control group-CG; CMS group-MG; Nifedipine group-NE; Dracocephalum moldavica L. low dosage group—DML·L; Dracocephalum moldavica L. middle dosage group -DML·M; Dracocephalum moldavica L. high dosage group -DML·H; WT-wall thickness; % WT-percentage of WT.

*Significantly different from the CG group, *P < 0.05, Δ significantly different from the MG group, Δ P < 0.05.

### Quantitative proteomics and bioinformatics analysis of CMS model rats and each intervention group

**Figure 3:**
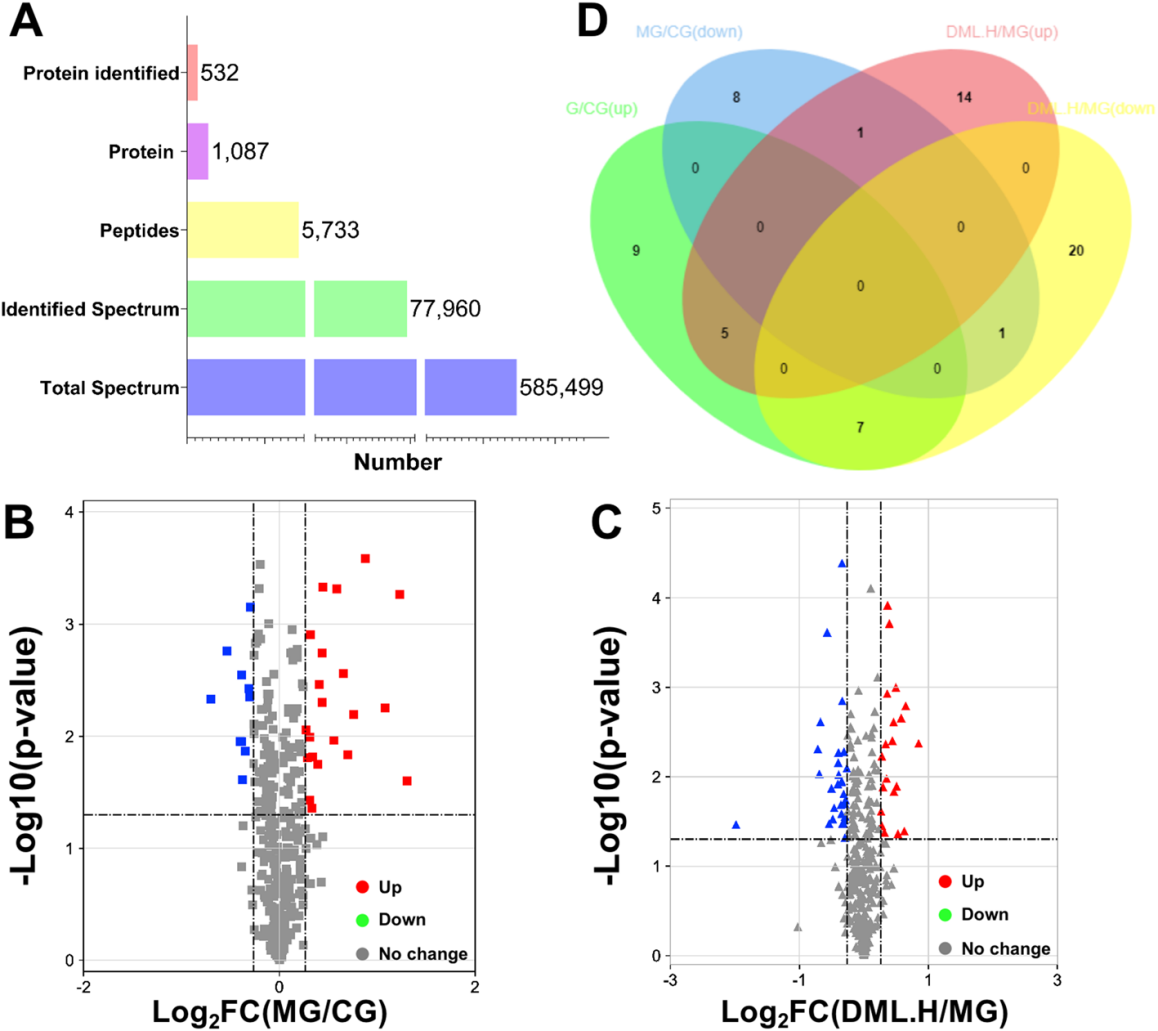
Quantitative proteomic analyses of a rat model of CMS. (**A**) Quality control validation of protein data. (**B**,**C**) Volcano plot of proteins expressed in the MG group, as compared to the CG and DML·H groups, respectively. The X-axis represents the log2-fold change of high (positive values) and low (negative values) expression levels of proteins in (**B**) the MG group compared to the CG group, and (**C**) the DML·H compared to the MG group. The Y-axis corresponds to the false discovery rate (FDR) of this fold change. Blue inverted triangles: proteins expressed at low levels with a FDR < 0.01; green inverted triangles: proteins expressed at low levels with a FDR < 0.05; black spot: proteins that did not show any significant difference in expression; red triangle: proteins expressed at high levels with a FDR < 0.01; yellow triangle: proteins expressed at high levels with a FDR < 0.05. (**D**) Venn-Euler diagrams of differentially expressed proteins in the MG group compared to the CG and DML·H groups, respectively.

In order to determine the protein expression profiles of each treatment group, we adopted iTRAQ technology. Figure 1 shows a schematic to depict how we used this technology. A total of 585,499 MS/MS spectra were obtained, matched to 77,960 secondary spectra, and 5733 different peptides were screened by cut-off value, corresponding to 1087 detected proteomes; 532 of these were considered to be proteins with a significant difference (as shown in Fig. 3A). The thresholds for DEPs were set to a fold change (FC) > 1.2 (up-regulated) or < 0.83 (down-regulated) and a *P* < 0.05 following statistical analysis. We exported the protein list to the ProteinPilot Descriptive Statistics Template (PDST) template to analyze the false discovery rate (FDR) and plotted this as a volcano map according to its logarithmic fold change (FC) and FDR value (Fig. 3B,C). Compared with rats in the CG group, rats in the MG group showed nine up-regulated proteins and eight down-regulated proteins in their serum samples. Compared with rats in the MG group, we detected 14 up-regulated proteins and 20 down-regulated proteins in serum samples from the DML·H group. Furthermore, between these two groups, we detected five co-upregulated proteins and one co-downregulated protein; seven proteins were up-regulated in the MG group and down-regulated in the DML·H group (as shown in Fig. 3D).

**Figure 4:**
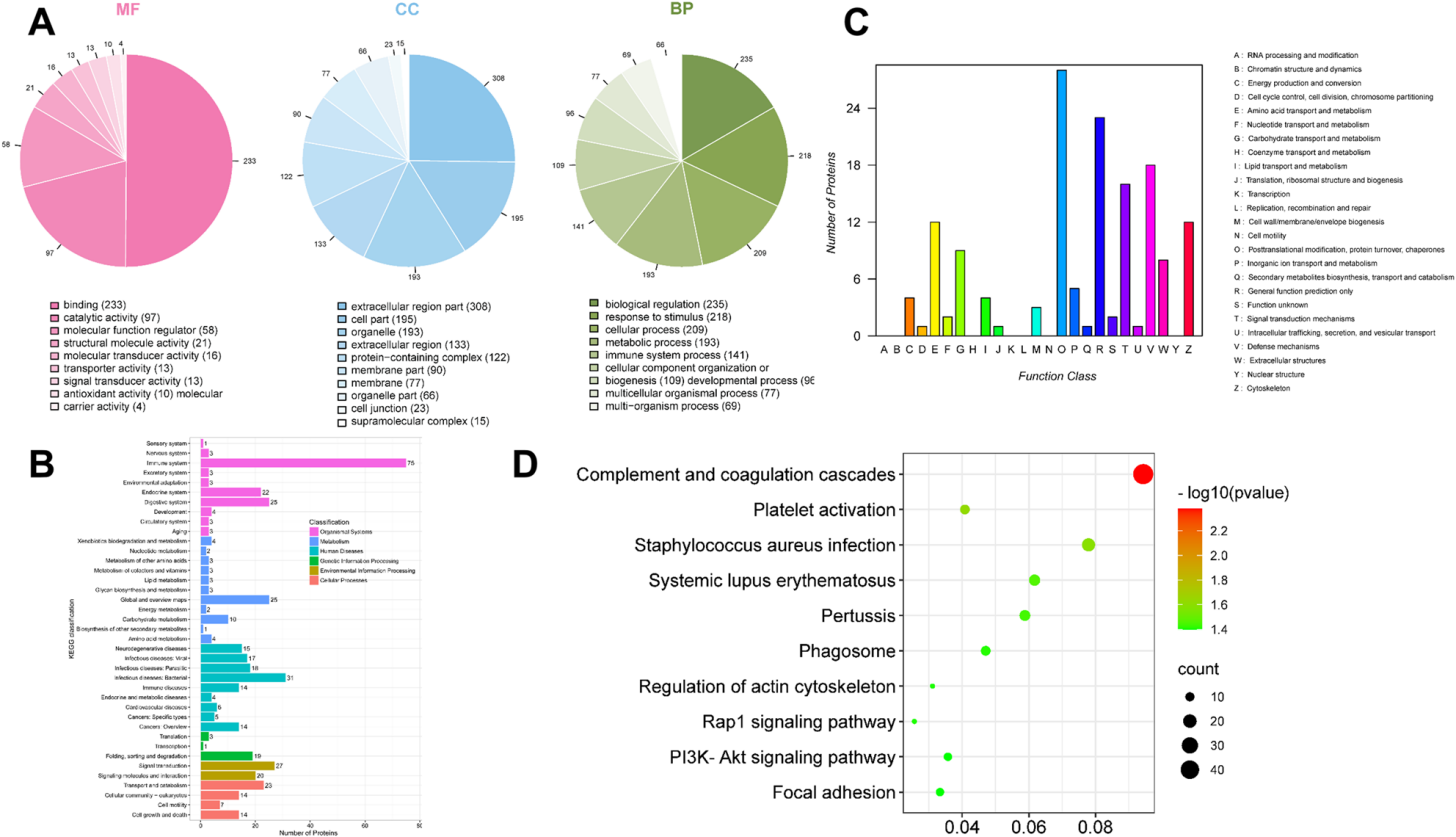
Quantitative bioinformatic analyses of a rat model of CMS. (**A**) GO functional annotation analysis of proteins identified by iTRAQ and categorized by biological process, molecular function, and cellular component, in the MG group as compared against the CG and DML·H groups. (**B**) KEGG pathway analysis of the proteins identified by iTRAQ. (**C**) COG function classification of the proteins identified by iTRAQ. (**D**)The top 20 pathways with the largest number of proteins.

In order to gain an overall functional view of the DEPs, we used gene ontology (GO) functional classification annotation and the Kyoto Encyclopedia of Genes and Genomes (KEGG) metabolic pathway analysis. Using GO analysis, we found that all of the proteins identified were classified according to their functions, in terms of biological process (BP), cell component (CC), and molecular function (MF), as shown in Fig. 4A–C. The molecular function enrichment distribution of the identified proteins was as follows: binding (233/465), catalytic activity (97/465), molecular function regulation (58/465), and structural molecular activity (16/465). The cell components showed enrichment and were distributed as follows: extracellular area part (308/1222), cell part (195/1222), organelle (193/1222). The distribution of biological process enrichment was as follows: biological regulation (235/1413), response to stimulation (218 /1413), cellular process (209/1413), and metabolic process (193/1413). KEGG pathway analysis (as shown in Fig. 4D) revealed the following distribution: coagulation and complement pathway (49/219), Staphylococcus aureus infection (19/219), and systemic lupus erythematosus (14/219). The functional classification and distribution of all identified proteins eukaryotic orthologous group (KOG) were as follows: post-transcriptional modification (28/150), general function prediction (23/150), and defense mechanism (18/150).

**Figure 5:**
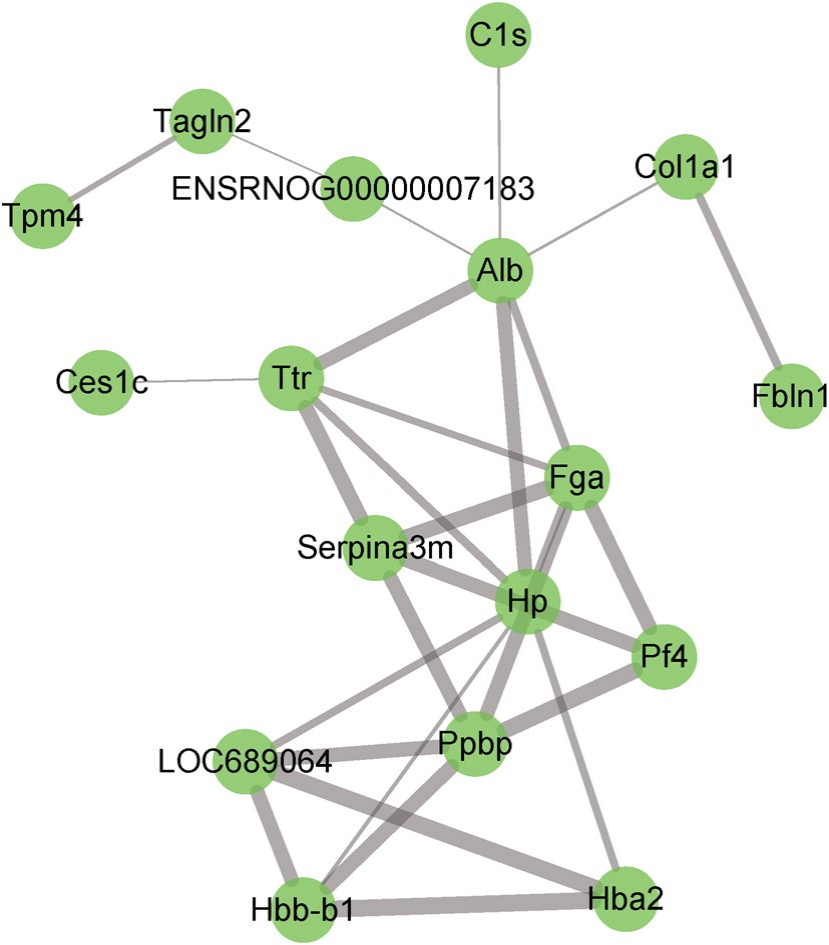
STRING analysis of the interactions between DEPs. α-1-acid glycoprotein, collagen, fibulin, haptoglobin, PLTP and TAGLN2 were identified at hub positions.

**Figure 6:**
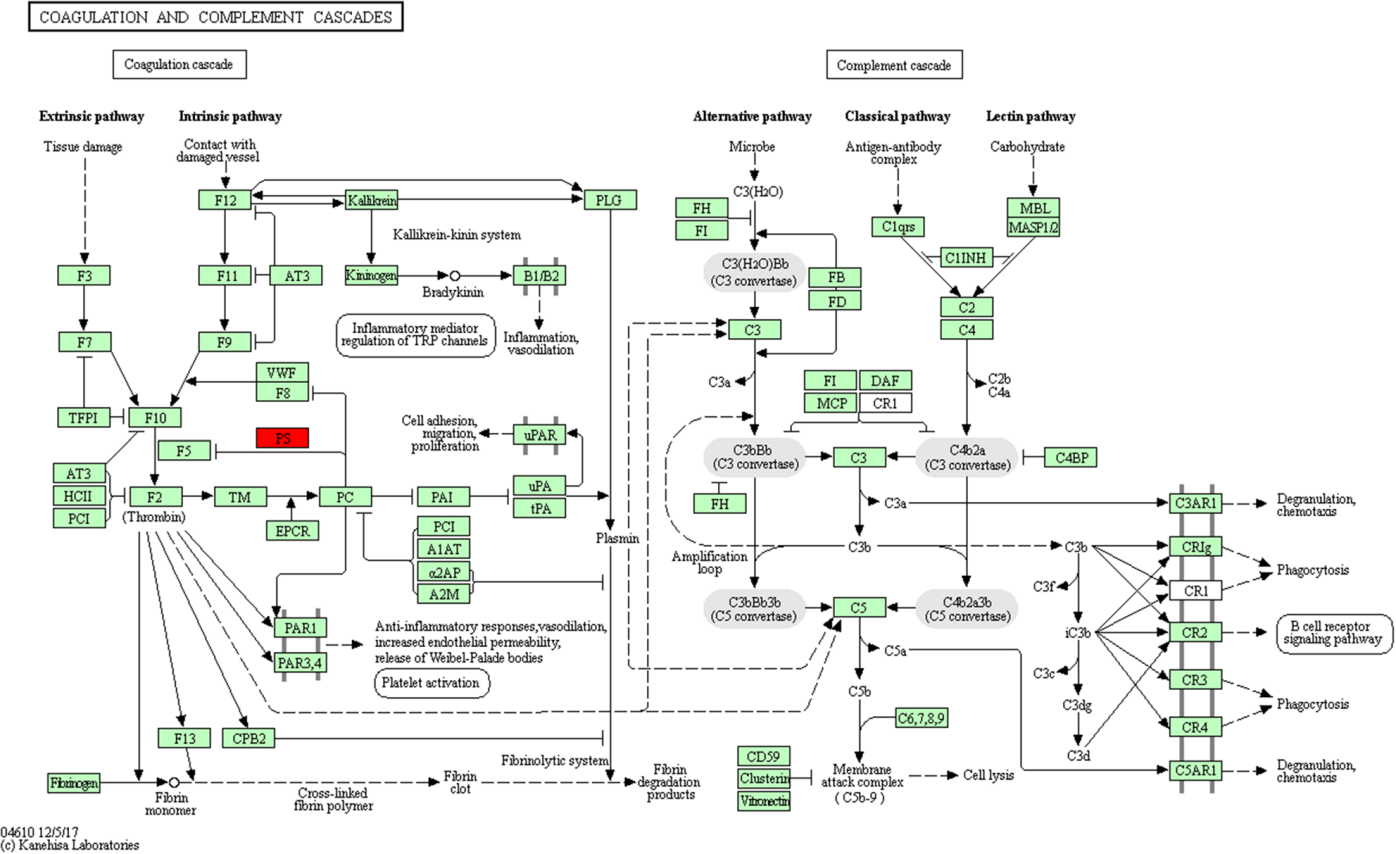
Network pathway of identified plasma proteins in the complement and coagulation cascades that were differentially abundant in CMS. Red and green squares denote proteins increased and decreased, respectively^36^.

In order to better understand the potential protein–protein interactions (PPIs), we used STRING software to perform PPI proteomic network analysis. This analysis identified six proteins that appear to play an essential role in the occurrence of CMS and the action of TFDM: α-1-acid glycoprotein, collagen, fibulin, haptoglobin, PLTP, and TAGLN2 (Fig. 5). GO and KEGG pathway analysis further revealed that the metabolic pathways associated with coagulation and complement are crucial to the occurrence of CMS through analysis of GO and KEGG pathways (Fig. 6).

### Immunohistochemical verification of DEPs

**Figure 7:**
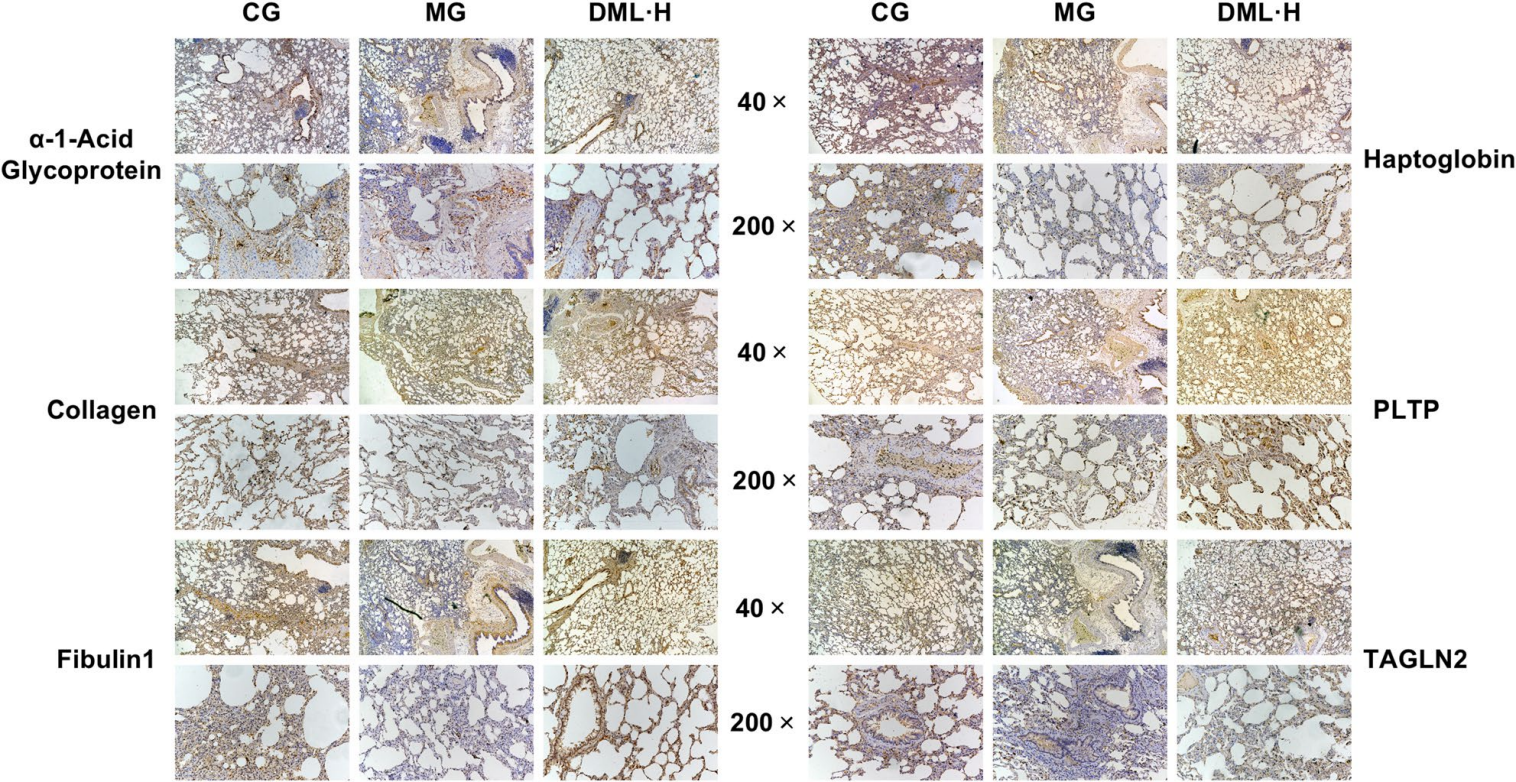
Immunohistochemical staining of DEPs. Positive immunostaining of α-1-acid glycoprotein, collagen, fibulin, haptoglobin, PLTP and TAGLN2 in CMS. These proteins were clearly suppressed in the DML·H group.

Immunohistochemical observation and analysis were performed on sections of lung tissue from each group of rats to verify the DEPs that were identified by iTRAQ proteomics. In lung tissue, α-1-acid glycoprotein, collagen, fibulin, haptoglobin, PLTP, and TAGLN2 are shown in yellow, as shown in Fig. 7. The levels of α-1-acid glycoprotein, collagen, fibulin, haptoglobin, PLTP, and TAGLN2 were significantly increased in the MG group. In contrast, the levels α-1-acid glycoprotein, collagen, fibulin, haptoglobin, PLTP, and TAGLN2 in the DML·H group were reduced. These results indicated that the expression levels of these protein were closely related to CMS and can be reversed by TFDM.

## Discussion

In the field of cardiovascular disease research, quantitative proteomics is mainly used to study and identify the pathophysiological mechanisms and developmental process underlying cardiovascular disease, involving congenital defects of heart development, atherosclerosis, hypertension, myocarditis, cardiomyopathy, myocardial infarction, arrhythmia, heart failure, and aneurysm. Over the past decade, proteomics technology has developed rapidly, including sample preparation, mass spectrometry, database searches, and bioinformatics technology, thus providing a complete set of objective protein tools for investigating cardiovascular disease. This technology has already made significant contributions to our understanding of the mechanisms associated with cardiovascular disease and clinical drug development^9^. On the other hand, the use of quantitative proteomics to investigate CMS is mainly targeted to the identification of new, specific, and sensitive DEPs for early diagnosis, early intervention of CMS, and pulmonary artery remodeling. Previously, an experimental animal model of CMS was successfully established. The quality control provided by iTRAQ labeling and subsequent bioinformatics analysis provided a highly appropriate foundation for us to obtain meaningful differential proteins.

### iTRAQ data identified candidate marker proteins for CMS

Proteomics research on CMS is mainly focused on the identification of early biomarkers and remodeling mechanisms after hypoxia in an attempt to develop effective diagnostic or therapeutic methods. Here, we identified 532 DEPs; several of these DEPs may be critical to CMS or pulmonary hypertension, including α-1-acid glycoprotein, collagen, fibulin, haptoglobin, PLTP, and TAGLN2. However, new candidates for the molecular changes involved in the occurrence of CMS may be determined by further investigation of these data. KEGG pathway analysis showed that all of the identified protein pathways are involved in coagulation and complement, pathways, Staphylococcus aureus infection, and systemic lupus erythematosus. We also performed GO enrichment analysis. Collectively, these analyses identified most of the biological processes affected by heart and lung dysfunction during CMS.

#### α-1-acid glycoprotein

Our analyses identified that the levels of α-1-acid glycoprotein in the MG group were significantly higher than those in the CG group. α-1-acid glycoprotein is an acute-phase protein that participates in a variety of immune mechanisms. α-1-acid glycoprotein can quickly and effectively control inflammatory damage and can exert defensive and protective functions. α-1-AGP is synthesized by the liver and can sensitively and quickly respond to tissue damage, inflammation, necrosis, and apoptosis, following stimulation by a variety of factors^10^.

#### Collagen

Our study showed that the pulmonary artery pressure was significantly higher in the MG group; pulmonary artery vasoconstriction is the key to the formation of pulmonary hypertension. The accumulation and cross-linking of collagen both play a role in the maintenance and deterioration of pulmonary artery pressure^11^. The amount of collagen in the lung tissue of rats in the MG group had increased significantly; this was because collagen content increased under hypoxic conditions, thus enhancing the remodeling and stiffness of the pulmonary artery vascular structure and therefore exacerbating the pulmonary hypertension. Previous studies have also found that when hypoxia, the amount of collagen is significantly higher than control group^12^. Collagen turnover can be significant during arterial remodeling.

#### Fibulin

Fibulin is a fibrinogen-binding blood protein and a component of many extracellular matrices (ECM), including blood vessels^13^. Fibulin can promote the activation of platelets in patients and become an essential indicator of lung injury and inflammation. Previous studies have demonstrated increased levels of fibulin in patients with chronic obstructive pulmonary disease (COPD) cigarette smoke-induced (CS-induced) experimental COPD in mice^14^. Pulmonary hypertension is also a common consequence of COPD^15^. Pulmonary hypertension is also one of the characteristics of CMS. In this study, we demonstrated that the MG group of rats had a significantly higher content of fibulin in the lungs of rats than that of the CG group. Consequently, fibulin is closely associated with the development of CMS and can be used as an auxiliary diagnostic sign.

#### Haptoglobin

Haptoglobin (Hp) is an acute-phase protein that scavenges hemoglobin in the event of intravascular or extravascular hemolysis. In humans, this protein exists in three main genotypes, Hp1-1, Hp2- 2, and Hp2-1^16^. Hp is involved in inflammatory reactions and has antibacterial, antioxidant, and angiogenic effects. Studies have shown that Hp is related to coronary heart disease, diabetes, hypertension, blood diseases, autoimmune diseases and malignant tumors. Hp also inhibits carbon monoxide and regulates the body's immune function^17^. The concentration of Hp in the serum will increase in a stressed state. During infection and tissue damage, Hp synthesis is significantly enhanced, thus reflecting the state of acute reaction^18^. This study showed that the levels of Hb protein in the lung tissue of rats in the MG group were significantly higher than those of the CG group. Therefore, Hp has value for diagnosis to CMS. Hp has been shown to participate in many life activities; consequently, bioinformatic research related to this protein is very important.

#### PLTP

Phospholipid transfer protein (PLTP) is a plasma protein that can regulate lipoprotein metabolism^19^. PLTP is a lipopolysaccharide-binding protein found in tissues and plasma. This protein can transport phospholipids and vitamin E between lipoproteins and cell membranes, and between lipoproteins, and is mainly associated with lipid metabolism^20^. However, aside from these processes, the physiological role of PLTP remains unknown. In humans and mice, the lungs are the dominant location for the expression of PLTP mRNA^21,22^. Previous studies have shown that PLTP might play an important role in maintaining the normal function of the lungs. In the lungs of human collagenase transgenic mice, an emphysematous animal model, the expression of PLTP mRNA was threefold higher than in control mice. However, the levels of mRNA in other tissues remained unchanged^23^. In this study, we demonstrated that the levels of PLTP protein in the lungs of rats in the MG group were significantly higher than those of rats in the CG group. Therefore, PLTP has value for diagnosis to CMS. We believe that PLTP is involved in lipid metabolism and a series of processes related to hypoxia. The specific mechanisms involved, however, require further study.

#### TAGLN2

TAGLN2, also known as Smooth Muscle22 alpha (SM22a), is a cytoskeletal protein. This protein is a differentiation marker of vascular smooth muscle cells (VSMC)^24^. TAGLN2 is abundantly expressed in differentiated VSMCs^25^. However, aside from this process, the physiological role of TAGLN2 in hypoxia remains unknown. Previous studies have shown that TAGLN2 binds to and colocalizes with F-actin. This protein participates in cytoskeleton organization and contraction regulation by combining with cytoskeleton actin^26^. This promotes remodeling and stiffness of the vascular structure in the pulmonary artery and exacerbates hypertension^27^. In our study, the expression of this protein was upregulated in the MG group; which may be due to pulmonary artery fibrosis and reduced pulmonary artery elasticity, thus leading to pulmonary hypertension.

### CMS and coagulation complement pathway

In this study, we demonstrated that the coagulation and complement metabolism pathways have been identified as a classical pathway. In general, the complement cascade is accompanied by interaction with the coagulation cascade^28^. High altitude hypoxia can reduce the function of the immune system, while neutrophils and macrophages can quickly adapt to the body's metabolic changes during hypoxia^29^. The effects of chronic hypoxia on the immune system mainly include T cells, B cells, natural killer cells, cytokines, and phagocytes^30^. However, the actual mechanism involved in the change of immune function under chronic hypoxia remains unclear, especially with regards to changes in the complement system under hypoxic stress. As a significant response of the immune system, the coagulation complement system can induce various mechanisms of immune regulation^31^ and play an important role in fighting infection. Previous studies have found that complement components C1, C4, C5, C6, C7, C8, C9, and complement factor H, are downregulated under hypoxic conditions at high altitude^30^. Animals lacking various complement components exhibit a range of manifestations related to host defense, including a greater susceptibility to infection, an impaired response to T cells and B cells, reduced phagocytic activity, and a reduced ability to remove pathogens and other immune complexes^32^. Therefore, we conclude that hypoxia at high altitude reduces the level of complement components in blood plasma.

### TFDM may improve pulmonary artery pressure and tissue morphology in CMS rats by reducing the expression of six DEPs

Compared with the CG group, the pulmonary artery pressure, the serum levels of Hb, and the Hct, in the MG group, NE group and TFDM group were significantly higher (*P* < 0.05). Compared with the MG group, the pulmonary artery pressure, the serum levels of Hb, and the Hct, in the NE group and the TFDM group were significantly reduced. The level of improvement was most significant in the DML·H group (*P* < 0.05). H&E staining showed that the pulmonary artery wall of the CG group was intact, the structure was normal, and the blood vessel wall was clear; the MG group showed the most significant morphological changes: the WT and %WT were significantly thicker, thus increasing pulmonary vascular resistance. Following the administration of TFDM, the morphology of the pulmonary artery tissue showed differing degrees of improvement; the most obvious improvement was observed in the TFDM low-dose group (DML·L). Expression levels of the six DEPs identified by iTRAQ were significantly elevated in the MG group but were significantly inhibited in the DML·H group; this was confirmed by immunohistochemistry. Therefore, we are of the opinion that TFDM may reduce the levels of these differential proteins to achieve vascular protection and reduce pulmonary artery pressure.

#### Clinical view

In this paper, we studied proteomic changes in a rat model of CMS and identified a series of proteins that may be considered as new biomarker candidates. Understanding the biological functions of these proteins can help clinicians make more effective diagnoses and design better treatments for CMS.

## Materials and methods

### Ethics statement

All of the experiments described herein were performed with the approval of the animal ethics committee of the First Affiliated Hospital of Xinjiang Medical University following the Care and Use of Laboratory Animals guidelines of the National Institutes of Health (Approval number: IACUC-20160218009). Further details are given in the Online Supplementary Material.

## Experimental design

Our animal study is reported in accordance with the ARRIVE guidelines for reporting experiments involving animals. Experiments were carried out in a randomized and blinded manner and statistical analyses were performed prior to revealing the treatment groups. Rats were assigned an identity number and assigned to groups randomly so that the experimenter was blinded to treatment.

### Materials

#### Animal

We purchased 120 healthy SD rats (8-weeks old) from the Experimental Animal Center of Xinjiang Medical University (male: 60; female: 60; weight: 160–200 g), animal license number: SCXK (Xinjiang) 2016-0003.

#### Instruments

Cryostat; Optical microscope(Leica); refrigerated centrifuge (Eppendorf); Vertical electrophoresis tank (Shanghai Tianneng Technology Co., Ltd. Shanghai, China); Constant temperature mixer (ABSON); Ultrasonic cell disruptor (Jiangsu Wavefield Intelligent Technology Co., Ltd.Jiangsu, China); Microplate reader softmax Pro (Molecular Devices); Automatic digital gel image analysis system (Shanghai Tianneng Technology Co., Ltd. Shanghai, China); Analytical balance (METTLER TOLED0); High performance liquid phase spectrometer (Waters); Series Mass spectrometer (Thermo Fishier Scientific), Northwest Special Environmental Artificial Test Chamber (Xinjiang Special Environmental Medicine Key Laboratory, located in Urumqi, Xinjiang). BC-5300Vet Automatic Hemacytometer and its ancillary reagents from WanRui Co., Shenzhen, China.

#### Pharmaceutical reagents

DML (produced in Xinjiang Madison Pharmaceutical Company Decoction Piece Factory, batch number: M30062307, Xinjiang, China); The extraction of TFDM: DML powder is added with ethanol (60%) at a solid-to-liquid ratio of 1:40, and refluxed and infiltrated at 50 °C. The total time is 50 min; Nifedipine tablets (produced by Shanxi Yunpeng Company, batch number: F160601, Shanxi, China); Ammonia, IAM iodoacetamide, Triethylammonium bicarbonate buffer, Trypsin protease, Sodium lauryl sulfate SDS, Acetone, BCA protein concentration Assay kit (enhanced), SDS-PAGE gel preparation kit, Coomassie brilliant blue staining solution (conventional method), iTRAQ 8 PLEX, Protein Ladder (non-prestained), Protease Inhibitor Cocktail, TCEP, Water LC/MS, Acetonitrile LC/MS, Methanol LC/MS, Formic acid LC/MS, ketamine, diazepam, atropine.

### Methods

#### Model building

The experimental conditions for rats in the control group (CG) were as follows: a simulated altitude of 720 m, a temperature range of 18–26 °C, a humidity range of 40–60%, a pressure of 93.2 kPa, and an oxygen partial pressure of 19.54 kPa. The control rats were fed under these conditions for 45 days with free access to food and no drug intervention. During the experiment, we made a series of observations relating to behavior, autonomous activities, food intake, drinking, hair, feces, urine, eyes, ears, nose, and mouth. We also checked for secretions and weighed each rat each day.

The experimental conditions for rats in the plateau model group (MG) were as follows: a simulated altitude of 5000 m, a temperature range of 18–26 °C, a humidity range of 40–60%, a pressure of 54.1 kPa, and an oxygen partial pressure of 10.84 kPa. The rats were fed for 30 days under this condition. During the experiment, we made a series of observations relating to behavior, voluntary activities, food intake, drinking, hair, feces, urine, eyes, ears, nose, and mouth; we also weighed each rat on a daily basis. The experiments were carried out in Northwest Special Environment Artificial Test Cabin, Xinjiang Special Environmental Medicine Key Laboratory, Urumqi, Xinjiang).

#### Experiment grouping and intervention measures

We randomly divided 120 rats into six groups. Using the stratified randomization method, we stratified the rats according to gender. We randomized models for 60 female rats and 60 male rats and a random ranking table was used to generate random numbers so that we could divide the rats into six groups. Finally, each group contained the same number of rats in each group; 20 rats in each group; half were male). The specific groupings were as follows:

- Plain control group (CG): No drug intervention was given and rats were kept in a plain environment for 45 days.
- Plateau model group (MG): No drug intervention was given and the rats were kept in a plateau environment for 45 days.
- Nifedipine group (NE): housed in a plateau environment for 45 days, treated with 2.7 mg/kg of nifedipine once a day on last 15 days.
- TFDM low-dose group (DML·L): housed in a plateau environment for 45 days, treated with 15 mg/kg of TFDM once a day on last 15 days.
- TFDM medium-dose group (DML·M): housed in a plateau environment for 45 days, treated with 30 mg/kg of TFDM once a day on last 15 days.
- TFDM high-dose group (DML·H): housed in a plateau environment for 45 days, treated with 60 mg/kg of TFDM once a day on last 15 days.

#### The determination of pulmonary artery pressure

The rats were anesthetized by intraperitoneal injection of 0.75 mL/100 g solution of atropine (0.5 mg/mL), ketamine (50 mg/mL), and diazepam (5 mg/mL). The rats were then fixed in the supine position, the neck and chest skin was cut and the thoracic cavity was opened along the midline of the sternum to expose the lungs and heart completely. An A7-gauge needle, filled with normal heparinized saline, was then inserted into the pulmonary artery to measure pulmonary artery pressure. The position of the needle was observed directly with the naked eye. The other end of the needle was connected to a pressure transducer with a self-made catheter, and a biosignal recorder was used to monitor pressure changes.

#### The determination of serum levels of Hb, Hct

Previous reports showed that the serum levels of Hb and the Hct were significantly increased in CMS rats^6^. Therefore, to verify that we had successfully established a rat model of CMS model, we measured the serum levels of Hb and the Hct by using the BC-5300 Vet Automatic Hemacytometer and ancillary reagents (WanRui Co., Shenzhen, China). The tests were completed within 0.5—1 h of blood sampling.

#### Pathological examinations of the lungs and pulmonary artery

Rats were euthanized 24 h after the final drug administration. (Rats were fixed on the lid of the rearing box, grasp the tail of the rat with one hand, pull it back with a slight force, and press the head with the thumb and index finger of the other hand quickly, use surgical scissors or the tweezers quickly pressed the rat’s neck, and both hands used force to dislocate the cervical spine, which caused the spinal cord and the brain to break.) The lungs were then removed and washed in normal saline. Lung tissues were then fixed with 10% formalin solution, dehydrated with a gradient series of ethanol, cleared with xylene, and embedded in paraffin. Tissues were then cut into 5 µm sections and stained with hematoxylin and eosin. We then used a light microscope to identify morphological changes in the cells and tissues. We also evaluated pulmonary arteries with diameters < 100 µm because previous reports showed that rats with pulmonary hypertension exhibited a significantly medial thickness in arteries that were < 100 µm in external diameter^33^. Vessel dimension was taken as the mean of two measurements made at right angles to each other. Wall thickness (WT) was estimated as the intima plus media; we also calculated the proportion of external diameter that was occupied by the WT (%WT) as follows: (2 × wall thickness)/external diameter × 100^34^.

#### Protein identification and quantification

Total protein was extracted from blood plasma with urea lysis buffer (7 M urea, 2 M thiourea, and 1% SDS) containing a protease inhibitor. Protein concentrations were then determined with a BCA Protein Assay Kit (Pierce, Thermo, USA). Following reduction, cysteine alkylation, and digestion, samples were then labeled with iTRAQ reagents (Applied Biosystems, 4390812) in accordance with the manufacturer's instructions. After being desalted with a C18 solid-phase extraction, peptides were then tested by Nano Liquid Chromatography-Mass Spectrometry/Mass Spectrometry analysis^35^.

RAW data files were analyzed using ProteomeDiscoverer (Thermo Scientific, Version 2.2) against the Rattus database (http://asia.ensembl.org). The MS/MS search criteria were as follows: a mass tolerance of 10 ppm for MS and 0.02 Da for MS/MS tolerance; trypsin as the enzyme with 2 missed cleavage events allowed; carbamido methylation of cysteine and the trimethyltin (TMT) of the N- terminus and lysine side chains of peptides as a fixed modification; and methionine oxidation as a dynamic modification. The false discovery rate (FDR) for peptide identification was set as ≤ 0.01. A minimum of one unique peptide identification was used to support protein identification.

#### GO function classification and KEGG metabolic pathway analysis

Annotation of all identified proteins was performed using GO (http://www.blast2go.com/b2ghome; http://geneontology.org/) and KEGG pathway (http://www.genome.jp/kegg/) analysis. DEPs were then used for GO and KEGG enrichment analysis. The functional network prediction of protein–protein interaction (PPI) provided by STRING database (version 11, http://string-db.org/)^36^.

#### Immunohistochemical detection

The separated lung tissue was embedded with O.C.T. and then quickly frozen in liquid nitrogen. Next, we prepared frozen sections (10 μm). After antigen retrieval, the sections were incubated at 4 °C overnight with primary antibodies against α-1-acid glycoprotein, collagen, fibulin, haptoglobin, PLTP, and TAGLN2. The next day, the sections were washed, and secondary antibodies were added dropwise. Sections were then incubated with diaminobenzidine (DAB), counterstained with hematoxylin, mounted with neutral gum, and images were acquired by microscopy.

#### Statistical analysis

The data were expressed as the mean ± standard deviation, with one-way ANOVA and Tukey's post hoc test used for multiple comparisons by SPSS (IBM, version 22.0), in which *P* < 0.05 was considered statistically significant.

## Supplementary Information

Supplementary Information 1.

Supplementary Information 2.

Supplementary Information 3.

## Data Availability

The datasets used or analyzed during the current study are available from the corresponding author on reasonable request.

## Abbreviations

CMS: Chronic mountain sickness
iTRAQ: Isobaric tags for relative and absolute quantification
DML: *Dracocephalum Moldavica* L.
TFDM: Total flavones of *Dracocephalum Moldavica* L.
DEPs: Differentially expressed proteins
CG: Plain control group
MG: Plateau model group
NE: Nifedipine group
DML·H: Total flavones of *Dracocephalum Moldavica* L. high-dose group
DML·M: Total flavones of *Dracocephalum Moldavica* L. medium-dose group
DML·L: Total flavones of *Dracocephalum Moldavica* L. low-dose group

## Code availability

All data, models, and code generated or used during the study appear in the submitted article.
